# A linear-time algorithm for reconstructing zero-recombinant haplotype configuration on a pedigree

**DOI:** 10.1186/1471-2105-13-S17-S19

**Published:** 2012-12-07

**Authors:** En-Yu Lai, Wei-Bung Wang, Tao Jiang, Kun-Pin Wu

**Affiliations:** 1 Institute of Biomedical Informatics, National Yang Ming University, Taipei 112, Taiwan; 2 Bioinformatics Program, Taiwan International Graduate Program, Academia Sinica, Taipei 115, Taiwan; 3 Department of Computer Science and Engineering, University of California, Riverside, CA 92521, USA

**Keywords:** Haplotype inference, zero-recombinant haplotype configuration (ZRHC), pedigree, mating loop.

## Abstract

**Background:**

When studying genetic diseases in which genetic variations are passed on to offspring, the ability to distinguish between paternal and maternal alleles is essential. Determining haplotypes from genotype data is called haplotype inference. Most existing computational algorithms for haplotype inference have been designed to use genotype data collected from individuals in the form of a pedigree. A haplotype is regarded as a hereditary unit and therefore input pedigrees are preferred that are free of mutational events and have a minimum number of genetic recombinational events. These ideas motivated the zero-recombinant haplotype configuration (ZRHC) problem, which strictly follows the Mendelian law of inheritance, namely that one haplotype of each child is inherited from the father and the other haplotype is inherited from the mother, both without any mutation. So far no linear-time algorithm for ZRHC has been proposed for general pedigrees, even though the number of mating loops in a human pedigree is usually very small and can be regarded as constant.

**Results:**

Given a pedigree with *n *individuals, *m *marker loci, and *k *mating loops, we proposed an algorithm that can provide a general solution to the zero-recombinant haplotype configuration problem in *O*(*kmn *+ *k*^2^*m*) time. In addition, this algorithm can be modified to detect inconsistencies within the genotype data without loss of efficiency. The proposed algorithm was subject to 12000 experiments to verify its performance using different (*n, m*) combinations. The value of *k *was uniformly distributed between zero and six throughout all experiments. The experimental results show a great linearity in terms of execution time in relation to input size when both *n *and *m *are larger than 100. For those experiments where *n *or *m *are less than 100, the proposed algorithm runs very fast, in thousandth to hundredth of a second, on a personal desktop computer.

**Conclusions:**

We have developed the first deterministic linear-time algorithm for the zero-recombinant haplotype configuration problem. Our experimental results demonstrated the linearity of its execution time in relation to the input size. The proposed algorithm can be modified to detect inconsistency within the genotype data without loss of efficiency and is expected to be able to handle recombinant and missing data with further extension.

## Background

A *genetic disease *is caused by the abnormality in an individual's genome. Genetic diseases have been studied extensively for decades by investigating the connection between diseases and genetic variations. In the human genome, chromosomes come in pairs; each gene consists of two *alleles *that reside in different chromosomes at the same locus. One of the two alleles comes from the father and the other comes from the mother. To study hereditary diseases in which the genetic variations are passed on to offspring, the ability to distinguish between paternal and maternal alleles is essential. Unfortunately, the *haplotype *structure of a human genome is not available directly from the genotyping and the unordered genotype data does not tell us which allele comes from which parent. A haplotype is a collection of alleles at multiple loci on a chromosome that tend to be inherited as a unit. The determination of haplotypes from genotype data is called *haplotype phasing *or *haplotype inference*. Algorithms for haplotype inference are indispensable and have been intensively studied.

**Figure 1:**
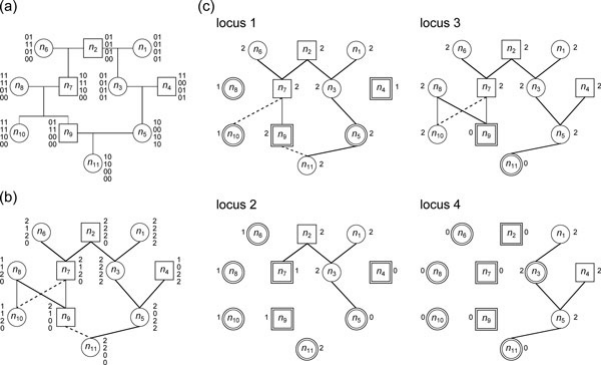
**A pedigree of 11 members**. (a) A pedigree of 11 members coupled with genotype data. The paternal haplotype of an individual is listed left while its maternal haplotype is listed right, even though the haplotype information is not available from genotyping. For example, the paternal and maternal haplotypes of individual *n*_6 _are 0100 and 1110, respectively; the genotype of *n*_6_, however, is specified as {0, 1}{1, 1}{0, 1}{0, 0}. Circles represent females and boxes represent males. Children are listed below their parents with line connections. For example, the couple *n*_7 _and *n*_8 _have two children *n*_9 _and *n*_10_. There is a mating loop in the pedigree due to the common ancestor *n*_2 _of the couple *n*_5 _and *n*_9_. (b) A pedigree graph with a spanning tree. Tree edges are solid lines and non-tree edges are dotted lines. The genotype data are represented as vectors of *g*-constant. There is a local cycle of length 4 due to the couple *n*_7 _and *n*_8 _and their children *n*_9 _and *n*_10_. There is a global cycle of length 6 due to the mating loop. (c) There are four locus graphs for the different loci. Edges in locus forests are depicted as solid lines. Nodes with thick borders are predetermined.

The existing computational algorithms for haplotype inference can be classified into statistical and combinatorial and most of which were designed for genotype data collected from individuals in the form of a *pedigree*. A pedigree is a hierarchical structure that describes the parent-child relationship among members of a family. Individuals without parents are called *founders*. There may be cycles in a pedigree, which are referred to as *mating loops*. A mating loop arises from a couple if they have children and both of them are offspring of certain family ancestors. An example of a pedigree, coupled with genotype data, is depicted in Figure 1(a); each allele is denoted as 0 or 1 to represent its form within a gene. If two alleles of a gene are the same, the locus is *homozygous*; otherwise, it is *heterozygous*. A haplotype is regarded as a hereditary unit and therefore an input pedigree is preferred to be free of mutational events and to have minimum number of genetic recombinational events [1]. Haplotype inference under this assumption is referred to as the *minimum-recombinant haplotype configuration (MRHC) *problem, which requires the solving of the haplotype structure of the input pedigree with the minimum number of recombination events [1]. Several algorithms have been proposed to solve the MRHC problem [1-8]. A special case of MRHC is *zero-recombinant haplotype configuration (ZRHC) *problem, which strictly follows the *Mendelian law of inheritance*, namely that one haplotype of each child is inherited from the father and the other haplotype is inherited from the mother, without any mutation [9]. To reduce the complexity of the ZRHC, some algorithms have been applied to pedigrees without mating loops (called *tree pedigrees*) [10-12]. In contrast to algorithms targeting tree pedigrees, so far no linear-time algorithm for ZRHC has been proposed for general pedigrees, even though the number of mating loops in a human pedigree is usually very small and can be regarded as constant; the execution time of existing algorithms for ZRHC using general pedigrees is polynomial [4,13-17]. Regardless of whether it is a MRHC or a ZRHC problem, some algorithms have been extended to handle pedigrees with mutations or missing data [5,8,11,15]. In addition to haplotype inference from pedigree data, algorithms have been proposed for *population *datasets that come from unrelated individuals. Algorithms for population datasets try to decode the haplotype structure of each individual as well as the haplotype frequencies of a population [18-22]. All the above mentioned algorithms are mainly combinatorial. Readers who are interested in statistical approaches for haplotype inference can consult a recent review [23].

In this study, we have targeted the ZRHC problem for pedigree data. If we assume we are given a pedigree with *n *individuals and *m *marker loci. Then for general pedigrees, Li and Jiang proposed an *O*(*m*^3^*n*^3^) time algorithm by converting the inheritance process into an equivalent linear system of *O*(*mn*) equations over Galois field GF(2) and invoking Gaussian elimination [4]. Xiao et al. improved the method to take *O*(*mn*^2 ^+ *n*^3 ^log^2 ^*n *log log *n*) time by removing redundant equations from the linear system [16]. Doan et al. proposed an *O*(*mnα*(*m*)) time algorithm by exploring constraints among marker loci rather than family members, where *α*(·) is the inverse of the Ackermann function [14]. For tree pedigrees, the execution time of the algorithm proposed by Xiao can be reduced from *O*(*mn*^2 ^+ *n*^3 ^log^2 ^*n *log log *n*) to *O*(*mn *+ *n*^3^) [16]. Li and Li proposed an *O*(*mnα*(*n*)) time algorithm using disjoint-set data structures [11]. Liu et al. further lowered the complexity of Xiao's algorithm to linear time *O*(*mn*) [12]. Chan et al. also proposed a linear-time algorithm by maintaining a graph structure [10]. Chan's algorithm, however, only produce a particular solution. A *particular solution *assigns a numerical value to each system variable, while a *general solution *describes all possible solutions of the system by designating certain variables as free variables and the others as linear combinations of these free variables.

In this paper, we presented an *O*(*kmn *+ *k*^2^*m*) time algorithm that provides a general solution for ZRHC for general pedigrees, where *k *is the number of mating loops. In human pedigrees, *k *is usually very small and can be regarded as constant. Our algorithm therefore turns out to be linear for most of the practical cases. The proposed algorithm was subject to 12000 experiments to verify its performance using different (*n, m*) combinations. The value of *k *was uniformly distributed between zero and six throughout all experiments. The experimental results show a great linearity of the execution time in relation to the input size when both *n *and *m *are larger than 100. For those experiments where *n *or *m *are less than 100, the proposed algorithm runs very fast, from thousandth to hundredth of a second on a personal desktop computer. We also showed that the proposed algorithm can be easily modified to detect inconsistencies among genotype data without loss of efficiency.

## Methods

To apply computational techniques, we transformed the input pedigree into a *pedigree graph *by connecting each parent directly to its children (Figure 1(b)). A pedigree graph is an undirected graph *G *= (*V, E*), where *V *is a set of nodes and *E *a set of edges. Each node in *V *represents an individual in the pedigree; each pair of nodes is connected with an edge in *E *if and only if the two individuals have a parent-child relationship. *G *is defined to be undirected because the computational property of each edge is symmetric in our algorithm, even if the parent-child relationship is asymmetric. *G *may contain cycles. We only pay attention to two types of cycles: a cycle due to a mating loop, which is called a *global cycle *and a cycle due to a couple and two of their children, which is called a *local cycle*. Global cycles and local cycles are referred to as *basic cycles*. For ease of cycle processing, we construct a spanning tree *T *(*G*) on *G*. A basic cycle can be obtained by adding a non-tree edge into *T *(*G*). The set of non-tree edges is denoted by *E^X^*. Non-tree edges are further divided into two disjoint subsets $E_{L}^{X}$ and $E_{G}^{X}$; members in $E_{L}^{X}$ induce local cycles and members in $E_{G}^{X}$ induce global cycles. Mating loops seldom appear in human pedigrees and therefore $|E_{G}^{X}|=k$ is regarded as a small constant.

In the rest of this paper, we are assuming that *G *has *n *nodes and *m *loci, all alleles are bi-allelic (denoted by 0 or 1), and the input dataset is free of genotyping errors. Under this assumption, the input size of ZRHC is *O*(*mn*). The genotype data of a node *n_i _*are represented as a vector $g_{n_{i}}$ of size *m*. The genotype of *n_i _*at locus *l*, where 1 ≤ *l *≤ *m*, is defined as follows:

$$g_{n_{i}}\left[l\right]=\left\{\begin{array}{cc} 0 & \text{if}\;\text{locus}\;l\;\text{is}\;\text{homozygous}\;\text{and both}\;\text{alleles}\;\text{are}\;{\text{0}}^{\prime }\text{s}\\ 1 & \text{if}\;\text{locus}\;l\;\text{is}\;\text{homozygous}\;\text{and both alleles}\;{\text{are 1}}^{\prime }s\\ 2 & \text{if}\;\text{locus}\;l\;\text{is}\;\text{heterozygous} \end{array}\right)$$

Genotype data are available, thus all *g*-variables can be regarded as constant (Figure 1(b)). We introduce a vector $p_{n_{i}}$ of size *m *to describe the haplotype information of *n_i_*; the paternal allele of *n_i _*at locus *l*, where 1 ≤ *l *≤ *m*, is defined as follows:

$$p_{n_{i}}\left[l\right]=\left\{\begin{array}{cc} 0 & \text{if}\;\text{paternal}\;\text{allele}\;\text{is}\;\text{0}\\ 1 & \text{if}\;\text{paternal}\;\text{allele}\;\text{is}\;\text{1}\text{.} \end{array}\right)$$

The vector $p_{n_{i}}$ is regarded as unknown even though we know that $p_{n_{i}}\left[l\right]=g_{n_{i}}\left[l\right]$ if *n_i _*is homozygous at locus *l *(i.e. $g_{n_{i}}$[*l*] ≠ 2).

We formulated the ZRHC problem as follows.

**ZRHC ***Given a pedigree graph G*(*V, E*) *with full g-constants, determine *$p_{n_{i}}$ of each node *n_i _*in *V*.

The haplotype configuration of the input pedigree is identified by specifying the paternal haplotype of each family member.

### A system of linear equations over GF(2)

In this section, we introduce a system of linear equations based on *G *and *g*-constants; this system was first proposed in [16] and will be reduced to determine all *p*-variables. Since *p*-variables carry binary values, all equations in the linear system are defined over GF(2) whose operations addition (+) and multiplication (·) are shown in Table 1.

**Table 1:** Addition (+) and multiplication (*·*) in GF(2)

+	0	1	*·*	0	1
0	0	1	0	0	0
1	1	0	1	0	1

#### The building block of the system: inheritance

"Inheritance" is the building block of the system. What parents pass to their children must be the same as what children receive from their parents. For a parent *n_i _*and a locus *l, n_i _*passes $p_{n_{i}}$[*l*] + 1 to its children if and only if the genotype of *n_i _*at locus *l *is heterozygous and *n_i _*passes its maternal allele; otherwise *n_i _*passes $p_{n_{i}}$ [*l*] to its children. We introduce two auxiliary variables $w_{n_{i}}\left[l\right]{\;\text{and}\;h}_{n_{i},n_{j}}$ to formally state the above argument. The variable $w_{n_{i}}\left[l\right]$ indicates if locus *l *of *n_i _*is heterozygous.

$$w_{n_{i}}\left[l\right]=\left\{\begin{array}{cc} 0 & \text{if}\;g_{n_{i}}\left[l\right]\neq 2\;\left(\text{i}.\text{e}.\;\text{homozygous}\;\text{at}\;\text{locus}\;l\right)\\ 1 & \text{if}\;g_{n_{i}}\left[l\right]=2\;\left(\text{i}.\text{e}.\;\text{heterozygous}\;\text{at}\;\text{locus}\;l\right) \end{array}\begin{array}{c} . \end{array}\right)$$

The variable $h_{n_{i},n_{j}}$ indicates which allele of *n_i _*is passed to its child *n_j_*.

$$h_{n_{i},n_{j}}=\left\{\begin{array}{cc} 0 & \text{if}\;n_{i}\;\text{passes}\;\text{its}\;\text{paternal}\;\text{allele}\;\text{to}\;n_{j}\\ 1 & \text{if}\;n_{i}\;\text{passes}\;\text{its}\;\text{maternal}\;\text{allele}\;\text{to}\;n_{j}. \end{array}\right)$$

Therefore, $p_{n_{i}}\left[l\right]+w_{n_{i}}\left[l\right]\cdot h_{n_{i},n_{j}}$ represents the allele at locus *l *that *n_i _*passes to *n_j_*.

On the other hand, assume that *n_j _*receives an allele from *n_i_*. If *n_i _*is *n_j_*'s father, what *n_i _*passes to *n_j _*is the paternal allele of *n_j_*. In this case, we have $p_{n_{i}}\left[l\right]+w_{n_{i}}\left[l\right]\cdot h_{n_{i},n_{j}}=p_{n_{j}}\left[l\right]$. If *n_i _*is *n_j_*'s mother, there are two sub-cases. If locus *l *of *n_j _*is homozygous, what *n_i _*passes to *n_j _*must be the same as the paternal allele of *n_j_*. In this case, we have $p_{n_{i}}\left[l\right]+w_{n_{i}}\left[l\right]\cdot h_{n_{i},n_{j}}=p_{n_{j}}\left[l\right]$. If locus *l *of *n_j _*is heterozygous, what *n_i _*passes to *n_j _*is the maternal allele of *n_j _*and is different from the paternal allele of *n_j_*. In this case, we have $p_{n_{i}}\left[l\right]+w_{n_{i}}\left[l\right]\cdot h_{n_{i},n_{j}}=p_{n_{j}}\left[l\right]+1$. The variable $w_{n_{j}}\left[l\right]$ can be used to indicate if locus *l *of *n_j _*is homozygous or heterozygous, the two sub-cases can therefore be combined into a single equation $p_{n_{i}}\left[l\right]+w_{n_{i}}\left[l\right]\cdot h_{n_{i},n_{j}}=p_{n_{j}}\left[l\right]+w_{n_{j}}\left[l\right]$. Moreover, if we introduce another auxiliary variable $d_{n_{i},n_{j}}\left[l\right]$ as follows,

$$d_{n_{i},n_{j}}\left[l\right]=\left\{\begin{array}{cc} 0 & \text{if}\;n_{i}\;\text{is}\;{n_{j}}^{\prime }\text{s}\;\text{father}\\ w_{n_{j}}\left[l\right] & \text{if}\;n_{i}\;\text{is}\;{n_{j}}^{\prime }\text{s}\;\text{mother}, \end{array}\right)$$

the inheritance relationship can be unified into the following equation:

(1) $$p_{n_{i}}\left[l\right]+w_{n_{i}}\left[l\right]\cdot h_{n_{i},n_{j}}=p_{n_{j}}\left[l\right]+d_{n_{i},n_{j}}\left[l\right]$$

Note that the *w*- and *d*-variables are constant by definition, and the *p*- and *h*-variables are unknowns. Equation (1) formulates the property of edge (*n_i_, n_j_*) in *G*: *p*-variables and *w*-constants are attributes of the nodes *n_i _*and *n_j_*, and *h*-variables and *d*-constants describe the inheritance relation associated with the edge (*n_i_, n_j_*). With the information provided by Equation (1), various constraints on *h*-variables can be generated by traversing different paths in *G*. Our algorithm was designed to first determine *h*-variables based on these constraints and then the solution to the ZRHC problem can be obtained by determining all *p*-variables based on the solved *h*-values and Equation (1). One point needs special care: if *n_j _*is a child of $n_{i},h_{n_{j},n_{i}}$ and $d_{n_{j},n_{i}}$ are undefined. In our algorithm, we make the *h*-variables and *d*-constants symmetrical such that $h_{n_{j},n_{i}}=h_{n_{i},n_{j}}$ and $d_{n_{j},n_{i}}=d_{n_{i},n_{j}}$.

#### Linear constraints on h-variables

To reduce the computational complexity of our algorithm, we try to make the number of unknowns in the coming linear system as small as possible. In the pedigree graph *G*, we have *mn p*-variables and at most 2*n h*-variables (since each individual has two parents and there are at most *n *individuals). Observe that if a node *n_i _*itself or one of its parents is homozygous at locus *l*, $p_{n_{i}}\left[l\right]$ is determined by definition and Equation (1). In this case *n_i _*is referred to as *predetermined *at locus *l *and the number of unknown *p*-variables is reduced by one. Moreover, for an edge (*n_i_, n_j_*) ∈ *E*, where *n_i _*is a parent of $n_{j},\;h_{n_{i},\;n_{j}}$ is cancelled from Equation (1) if $w_{n_{i}}\left[l\right]=0$ at locus *l*. If $w_{n_{i}}\left[l\right]=0$ holds for all 1 ≤ *l *≤ *m*, no constraints are imposed on $h_{n_{i},n_{j}}$ and it becomes a free variable (or its value will finally depend on other free variables). In this case the number of *h*-variables to be determined is reduced by one, which is equipotent to the removal of edge (*n_i_, n_j_*) from *G*. Accordingly, *w*-constants can be viewed as the weight of edges in *G*; we only pay attention to edges with weight one (parent nodes that are heterozygous). To consider only the edges with weight one at locus *l*, we construct the *lth locus graph G_l _*= (*V, E_l_*), where *E_l _*= {(*n_i_, n_j_*) | *n_i _*is a parent of *n_j_*, $w_{n_{i}}\left[l\right]=1$}. Moreover, the spanning forest *T*(*G*) ∩ *G_l _*is denoted by *T*(*G_l_*) and is referred to as the *lth locus forest *(Figure 1(c)).

We define constraints on *h*-variables by traversing paths in the locus graphs. Consider a path *p *= *n*_0_, *n*_1_, ..., *n_i _*in *G_l_*. Assume that *n*_0 _and *n_i _*are predetermined and all other in-between nodes are non-predetermined. Adding up all *h*-variables on the path will produce the following equation by Equation (1):

(2) $$\overset{i-1}{\underset{j=0}{\sum }}h_{n_{j},n_{j+1}}=p_{n_{0}}\left[l\right]+p_{n_{i}}\left[l\right]+\overset{i-1}{\underset{j=0}{\sum }}d_{n_{j},n_{j+1}}\left[l\right]=b.$$

Since *n*_0 _and *n_i _*are predetermined and all *d*-constants are known, *b *is a constant. The constant *b *is said to be the constraint of path *p*. Note that the constraint *b *does not depend on the direction that path *p *is read because the *h*-variables and *d*-constants are symmetric. Moreover, if the path is a cycle *c *= *n*_0_, *n*_1_, ..., *n_i_, n*_0 _in *G_l_*, we would have the following equation:

(3) $$\overset{i}{\underset{j=0}{\sum }}h_{n_{j},n_{j+1\text{mod}\;i+1}}=\overset{i}{\underset{j=0}{\sum }}d_{n_{j},n_{j+1\text{mod}\;i+1}}\left[l\right]=b^{\prime }.$$

Again, since all *d*-constants are known, *b*' is also a constant. The constant *b' *is said to be the constraint of cycle *c*. On the basis of Equations (2) and (3), we can generate constraint equations with only *h*-variables for cycles or for paths that connect predetermined nodes in *G_l_*. Constraints can be classified into two categories with respect to the spanning tree *T*(*G*): *cycle and path constraints *derived from paths containing non-tree edges, and *tree constraints *derived from paths containing only tree edges.

#### Cycle and Path constraints

Adding a non-tree edge *e *into the spanning tree *T *(*G*) generates a basic cycle *c*. If *G_l _*contains *e*, there are two cases of *c *in *G_l_*.

**Case 1 ***c *is in *G_l_*. A *cycle constraint b_c _*of cycle *c *can be obtained by Equation (3). The constraint is denoted interchangeably by *b_c _*or (*b_c_, e*), which is also said to be the cycle constraint of *e*.

**Case 2 ***c *is broken into several disjoint paths in *G_l _*by predetermined nodes. Since these paths are disjoint, there is exactly one path *p' *of them containing *e*. Along the path *p', *we identify a subpath *p *= *n_i _*...*n_j _*containing *e *such that *n_i _*and *n_j _*are predetermined and all other in-between nodes are non-predetermined. A *path constraint b_p _*of the subpath *p *can be obtained by Equation (2). The constraint is denoted interchangeably by *b_p _*or (*n_i_, n_j_, b_p_, e*), which is also said to be the path constraint of *e*. Path constraints are symmetric because (*n_i_, n_j_, b_p_, e*) = (*n_j_, n_i_, b_p_, e*).

#### Tree constraints

For each connected component of *T *(*G_l_*), we arbitrarily pick a predetermined node *n_s _*as the seed. For the unique tree path *p *that connects *n_s _*and another predetermined node *n_k _*in the same connected component, a *tree constraint b_t _*of path *p *can be obtained by Equation (2). The constraint is denoted interchangeably by *b_t _*or (*n_s_, n_k_, b_t_*). Tree constraints are symmetric because (*n_s_, n_k_, b_t_*) = (*n_k_, n_s_, b_t_*). Note that if there exists a component that has no predetermined nodes, locus *l *must be heterozygous across the entire pedigree and no tree constraints will be generated.

### Our algorithm in relation to the ZRHC problem

Our algorithm consists of four steps. We begin by initializing required data structures in the *preprocessing *step. The initialized data structures are subject to the *constraint generation *step to construct a system of linear constraints on *h*-variables. There are two issues should be addressed. First, since all constraints are derived from locus graphs that come from the same pedigree graph, there is usually redundancy in the system. Second, we actually do not need to know all *h*-values to solve the ZRHC problem. For a child node *n_i_*, there are two *h*-variables related to it and its parents. However, from Equation (1) we know that one of the two *h*-values is sufficient to determine $p_{n_{i}}$. So it is easy to see that the (*n *- 1) *h*-variables in *T *(*G*) form a minimal sufficient set to solve the ZRHC problem. In the third step, *constraint reduction and transformation*, we therefore try to eliminate redundancy in the system and transform as many path constraints into tree constraints as possible. Finally, in the *haplotype determination *step, we introduce an efficient way to solve *h*-variables and further *p*-variables based on the reduced system.

#### Step 1: preprocessing

The data structures of our algorithm are initialized by the following procedures:

1. Transform the pedigree into a pedigree graph *G *= (*V, E*). Each node *n_i _*in *V *is equipped with its genotype vector $g_{n_{i}}$. Since each individual has two parents, there are at most 2*n *edges in *G*, so we have |*V*| = *O*(*n*) and | *E *| = *O*(*n*).

2. Construct a spanning tree *T *(*G*) on *G*.

3. For each locus *l*,

(a) generate a locus graph *G_l_*,

(b) generate a locus forest *T *(*G_l_*), and

(c) identify predetermined nodes as well as their *p*-values, all *d*-constants, and all *w*-constants.

The operations applied in this step are graph traversal and spanning tree construction, both operations can be performed in time *O*(| *V *| + | *E *|) = *O*(*n*). The time complexity of this step is therefore *O*(*mn*).

#### Step 2: constraint generation

A system of linear equations on *h*-variables over GF(2) will be constructed in this step. The system consists of three sets *C^C^, C^P^*, and *C^T ^*that contain different kinds of constraints. *C^C ^*contains cycle constraints, if any, of all non-tree edges at all loci. Similarly, *C^P ^*contains path constraints, if any, of all non-tree edges at all loci. Finally, *C^T ^*contains tree constraints at all loci. To reduce computational complexity, repetitions of set members are forbidden in our algorithm; we do nothing if an existing member is going to be added into the same set.

There are *O*(*mn*) trials to generate a constraint for a non-tree edge since there are *m *locus graphs and each of which contains *O*(*n*) non-tree edges; in each trial we perform a cycle detection procedure to generate a cycle constraint or a path constraint, so we have | *C^C ^*| + | *C^P ^*| = *O*(*mn*). The cycle detection procedure is usually implemented by depth first graph traversal and its execution time depends on the length of the cycle. Consequently, if a non-tree edge induces a global cycle, the cycle detection procedure takes *O*(*n*) time; otherwise the procedure takes constant time because each local cycle contains only four edges. The time to generate *O*(*mn*) cycle and path constraints is *O*(*kmn*) since there are at most *km *trials to generate global cycle constraints. To generate tree constraints within a locus graph, we perform tree traversal on its locus forest. This procedure generates *O*(*n*) tree constraints in *O*(*n*) time. So we require *O*(*mn*) time to generate tree constraints at all loci. The time complexity to generate our constraint system is therefore *O*(*kmn*) + *O*(*mn*) *= O*(*kmn*).

#### Step 3: constraint reduction and transformation

Redundancy arises in the constraint system if a constraint can be represented as a linear combination of other constraints. We are especially interested in the following two types of redundancies.

**Figure 2:**
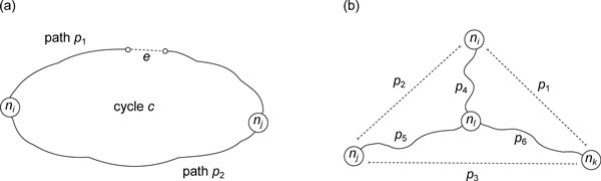
**Two types of redundancy arise from linearly dependence**. (a) A cycle *c *is decomposed into a tree path *p*_2 _and a path *p*_1 _that contains a non-tree edge *e*. So we have *b_c _*= *b_p _*+ *b_t_*, where *b_c_, b_p_*, and *b_t _*are constraints of cycle *c*, path *p*_1 _and path *p*_2_, respectively. The dotted line represents the non-tree edge *e*. (b) *n_l _*is the node closest to *n_i _*on the path *p*_3_. Assume that the constraint of tree path *p_i _*is *b_i_*, 1 ≤ *i *≤ 6. We have *b*_1 _= *b*_4 _+ *b*_6_, *b*_2 _= *b*_4 _+ *b*_5_, and *b*_3 _= *b*_5 _+ *b*_6_, which conclude that *b*_1 _= *b*_2 _+ *b*_3 _due to the addition over GF(2).

**Type 1 **Assume there is a basic cycle *c *in *G *and it can be decomposed into two edge-disjoint paths *p*_1 _and *p*_2 _both connecting nodes *n_i _*and *n_j_*. There must be exactly a non-tree edge *e *in *c*, and without loss of generality, we assume that *e *belongs to path *p*_1_. If there is a cycle constraint (*b_c_, e*) of *c*, a path constraint (*n_i_, n_j_, b_p_, e*) of *p*_1_, and a tree constraint (*n_i_, n_j_, b_t_*) of *p*_2_, we have *b_c _*= *b_p _*+ *b_t _*by Equations (2) and (3). That is, these three constraints are linearly dependent and each of them can be represented as a linear combination of the other two constraints (Figure 2(a)). A path constraint can therefore be transformed into a tree constraint by the equation *b_t _*= *b_p _*+ *b_c_*, which is the basis of the reduction of our constraint system.

**Type 2 **Assume there are three tree constraints (*n_i_, n_j_, b*_1_), (*n_i_, n_k_, b*_2_), and (*n_j_, n_k_, b*_3_) of paths *p*_1_, *p*_2_, and *p*_3_, respectively. By definition we know that a tree constraint is the summation of all *h*-variables along a unique path in *T*(*G*), so we have

$$\left\{\begin{array}{c} \begin{array}{c} b_{1}=\underset{\left(n_{x,}n_{y}\right)\in p_{1}}{\sum }h_{n_{x},n_{y}}\\ b_{2}=\underset{\left(n_{x},n_{y}\right)\in p_{2}}{\sum }h_{n_{x,}n_{y}} \end{array}\\ b_{3}=\underset{\left(n_{x,}n_{y}\right)\in p_{3}}{\sum }h_{n_{x,}n_{y}}. \end{array}\right)$$

Suppose that *n_l _*is the node closest to *n_i _*on the path *p*_3_. We then have three paths *p*_4 _between *n_i _*and *n_l_, p*_5 _between *n_l _*and *n_j_*, and *p*_6 _between *n_l _*and *n_k _*such that *p*_1 _= *p*_4 _+ *p*_5_, *p*_2 _= *p*_4 _+ *p*_6_, and *p*_3 _= *p*_5 _+ *p*_6_. The tree constraints can therefore be rewritten as

$$\left\{\begin{array}{c} b_{1}=\underset{\left(n_{x,}n_{y}\right)\in p_{4}}{\sum }h_{n_{x,}n_{y}}+\underset{\left(n_{x,}n_{y}\right)\in p_{{}_{5}}}{\sum }h_{n_{x,}n_{y}}\\ b_{2}=\underset{\left(n_{x,}n_{y}\right)\in p_{4}}{\sum }h_{n_{x,}n_{y}}+\underset{\left(n_{x,}n_{y}\right)\in p_{6}}{\sum }h_{n_{x,}n_{y}}\\ b_{3}=\underset{\left(n_{x,}n_{y}\right)\in p_{5}}{\sum }h_{n_{x,}n_{y}}+\underset{\left(n_{x,}n_{y}\right)\in p_{6}}{\sum }h_{n_{x,}n_{y}}\cdot \end{array}\right)$$

Because all constraints are defined over GF(2), we conclude that *b*_1 _+ *b*_2 _= *b*_3_; the three tree constraints are linearly dependent and each of them can be represented as a linear combination of the other two constraints (Figure 2(b)). The above argument implies the following lemma.

**Lemma 1 ***For any three nodes n_i_, n_j_, and n_k_, the tree constraint of the path between n_j _and n_k _is equal to the total tree constraint of the path between n_i _and n_j _and the path between n_i _and n_k_*.

Lemma 1 still holds even if *n_i _*is on the path between *n_j _*and *n_k _*(*n_i _*= *n_l _*in Figure 2(b)), which means that if a tree path is partitioned into two disjoint sub-paths, the tree constraint of this path is equal to the total constraint of the two sub-paths.

In this step, we remove the type 1 redundancy by transforming as many path constraints to tree constraints as possible, and remove the type 2 redundancy by reducing *C^T ^*to an equivalent set whose cardinality is at most (*n *- 1).

For each non-tree edge *e *∈ *E^X^*, if cycle constraint (*b_c_, e*) exists, we remove all path constraints (*n_i_, n_j_, b_p_, e*), if any, from *C^P ^*and add tree constraints (*n_i_, n_j_, b_c _*+ *b_p_*) into *C^T^*. Since the size of *C^P ^*is *O*(*mn*), this procedure can be carried out in time *O*(*mn*), and the new *C^T ^*is of size *O*(*mn*).

**Figure 3:**
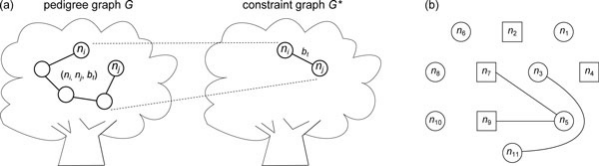
**The concept of a constraint graph**. (a) A tree constraint (*n_i_, n_j_, b_t_*) of the path that connects *n_i _*and *n_j _*in a pedigree graph *G *will be transformed into an edge between *n_i _*and *n_j _*with weight *b_t _*in the corresponding constraint graph G*. (b) A constraint graph. There are three edges (*n*_3_, *n*_11_), (*n*_7_, *n*_5_), and (*n*_9_, *n*_5_) in the constraint graph, which means that there are three tree constraints in the linear system. Note that the constraint graph is disconnected and contains several connected components.

To further remove the redundancy in *C^T^*, we construct a constraint graph G* of *G*. The constraint graph G* shares the same set of nodes *V *as *G*; for each tree constraint (*n_i_, n_j_, b_t_*) ∈ *C^T^*, we introduce an edge connecting nodes *n_i _*and *n_j _*in G* with weight *b_t _*(Figure 3(a)). An example of constraint graph is depicted in Figure 3(b). The constraint graph is used to reduce the size of *C^T^*. As shown in Figure 3(b), a constraint graph may not be connected. Within each connected component in G*, we randomly choose a seed *n_s _*and try to assign each node *n_i _*a variable *W*[*n_i_*] to represents the tree constraint of the tree path between nodes *n_s _*and *n_i _*in the pedigree graph *G*. The assignment is carried out by the following steps in each connected component of G*.

1. *W*[*n_s_*] of the seed *n_s _*is assigned the value zero,

2. start from *n_s_*, perform a breadth-first-search traversal via tree constraints, i.e., we can traverse from node *n_i _*to node *n_j _*if (*n_i_, n_j_, b_t_*) ∈ *C^T ^*or (*n_j_, n_i_, b_t_*) ∈ *C^T^*,

3. as we traverse from *n_i _*to *n_j _*through (*n_i_, n_j_, b_t_*) or (*n_j_, n_i_, b_t_*), if *n_j _*is unvisited, we assign *W*[*n_i_*] + *b_t _*to *W*[*n_j_*] based on Lemma 1; otherwise we do nothing.

Since *W*[*n_i_*] represents the tree constraint (*n_s_, n_i_, W*[*n_i_*]), it can be regarded as the summation of *h*-variables along the unique path on *T*(*G*) from the seed *n_s _*to node *n_i_*, which implies the following lemma:

**Lemma 2 **The h-value of a tree edge (n_i_, n_j_) in T(G) can be obtained by $h_{n_{i},n_{j}}=W\left[n_{i}\right]+W\left[n_{j}\right]$ if n_i _and n_j _reside in the same connected component of G*.

Therefore, if we can assign *W*-values to all nodes in *V *and make G* connected, G* would be equipotent to a reduced *C^T ^*of size (*n *- 1) that covers *h*-variables of all tree edges of *T*(*G*) and is sufficient to solve the ZRHC problem. The construction of the constraint graph takes *O*(|*C^T^*|) = *O*(*mn*) time.

The constraint graph G*, however, may not be connected with fully assigned *W*-values. We therefore introduced an extension procedure to extend G* by adding extra tree constraints, if any, into G*; we would like to reduce the number of connected component in G* as much as possible. To explore more tree constraints to be added into G*, we examine those non-tree edges *e *∈ *E^X ^*that do not have cycle constraints in *C^C^*. The basic idea is that if we can synthesize a new cycle whose constraint is the same as the expected cycle constraint of *e*, we may obtain new tree constraints by transforming known path constraints of *e*.

**Figure 4:**
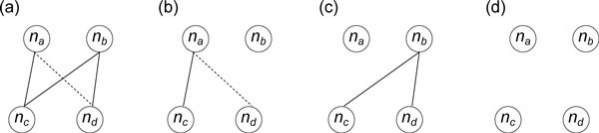
**All possible appearances of a local cycle in a locus graph**. The dotted line represents the non-tree edge *e*. (a) The local cycle appears with cycle constraint (*b_c_, e*). (b) There is only one path containing *e *with path constraint (*n_c_, n_d_, b_p_, e*). (c) There is only one path with tree constraint (*n_c_, n_d_, b_t_*). (d) There are only four predetermined nodes without any constraint.

For a non-tree edge *e* without cycle constraint, we try to synthesize a cycle only if $e\in E_{G}^{X}$. We do nothing if $e\in E_{L}^{X}$ because no extra tree constraints of *e *can be obtained by cycle synthesis. To see this, suppose the local cycle induced by *e *connects a couple *n_a _*and *n_b _*and their two children *n_c _*and *n_d_*; without loss of generality, we assume *e *= (*n_a_, n_d_*) (Figure 4). We can examine the possible constraints derived from this local cycle. Constraints of a single edge with predetermined endpoints are not of interest and can be ignored because the *p*-values of the endpoints are known; we need only pay attention to constraints whose path lengths are longer than one. In the *l*th locus graph, if $w_{n_{a}}\left[l\right]=w_{n_{b}}\left[l\right]=1$, a local cycle exists and we have cycle constraint (*b_c_, e*) (Figure 4(a)); if $w_{n_{a}}\left[l\right]=1$ and $w_{n_{b}}\left[l\right]=0$, we only have the path *p*_1 _= *n_c_n_a_n_d _*with path constraint (*n_c_, n_d_, b_p_, e*) (Figure 4(b)); if $w_{n_{a}}\left[l\right]=0$ and $w_{n_{b}}\left[l\right]=1$, we only have the path *p*_2 _= *n_c_n_b_n_d _*with tree constraint (*n_c_, n_d_, b_t_*) (Figure 4(c)); if, $w_{n_{a}}\left[l\right]=w_{n_{b}}\left[l\right]=0$ all four nodes are predetermined and we can determine their *p*-values directly (Figure 4(d)). No useful constraints other than (*b_c_, e*), (*n_c_, n_d_, b_p_, e*), and (*n_c_, n_d_, b_t_*) can be derived from this local cycle. Here we already know that (*b_c_, e*) does not exist. If (*n_c_, n_d_, b_t_*) is already in *C^T^*, it is the only useful tree constraint of *e *and we are finished. If (*n_c_, n_d_, b_t_*) does not exist in *C^T^*, we cannot obtain (*n_c_, n_d_, b_t_*) by combining *b_c _*and *b_p _*because (*b_c_, e*) does not exist, even if the path constraint (*n_c_, n_d_, b_p_, e*) is available. If this case holds for all 1 ≤ *l *≤ *m*, our linear system actually provides no information to obtain the tree constraint of *p*_2_; the *h*-variable of each edge on *p*_2 _will eventually be assigned a free variable, or its value will depend on other free variables. Therefore we do nothing if $e\in E_{L}^{X}$.

Assume that *E^S ^*is the set of non-tree edges in $E_{G}^{X}$ without cycle constraint. Cycle synthesis is carried out by concatenating paths with known path constraints or tree constraints. The extension procedure is applied to *E^S ^*as follows.

**Figure 5:**
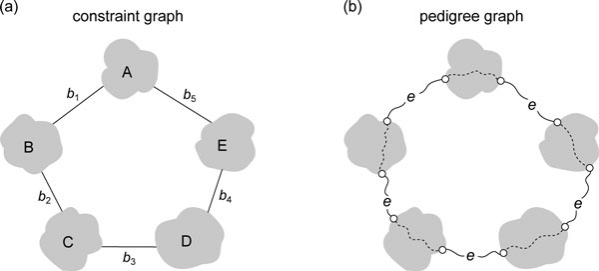
**The concept of a synthetic cycle**. (a) Five path constraints *b*_1_, *b*_2_, ..., *b*_5 _link five connected components A, B, C, D, and E to form a synthetic cycle in a constraint graph. (b) The conceptual view of the synthetic cycle of (a) in a pedigree graph. The synthetic cycle is actually a round trip through the tree edges and the non-tree edge *e*. The trip is composed of 10 different but connected paths in the pedigree graph. In this example, *e *would be visited five times during the trip.

E1. For each *e *∈ *E^S^*, we check if there is an odd number, say 2*t *+ 1, of path constraints of *e *that link different connected components in G* to form a *synthetic cycle *(Figure 5(a)); a constraint is said to link two components *A *and *B *if one of its endpoints resides in *A *and the other resides in *B*. There is a special case whereby we can also obtain a synthetic cycle if two endpoints of a single path constraint reside in the same connected component (*t *= 0). If no such 2*t *+ 1 path constraints are found, we cannot synthesize a cycle of *e *and do nothing; otherwise we perform the following tasks:

E1.1 assign the constraint $\left(\hat{b_{c}},e\right)$ to the synthetic cycle, where

(4) $$\hat{b_{c}}=\underset{\left(n_{i},n_{j},b_{p},e\right)\in S_{e}}{\sum }W\left[n_{i}\right]+W\left[n_{j}\right]+b_{p},$$

in which *S_e _*is the set of the chosen 2*t *+ 1 path constraints;

E1.2 for each path constraint $\left(n_{x},n_{y},b_{p}^{\prime },e\right)$ in *C^P^*, generate a tree constraint $\left(n_{x},n_{y},\hat{b_{c}}+b_{p}^{\prime }\right)$ and add the new constraint into G*;

E1.3 update G*;

E1.4 remove *e *from *E^S^*;

E2. If *E^S ^*becomes empty (there has been a synthetic cycle for every *e *in the original *E^S^*), or no synthetic cycle is synthesized (*E^S ^*stays unchanged), we stop the extension procedure; otherwise we go back to E1 to start the next iteration.

We thus try to synthesize a cycle for each non-tree edge in *E^S ^*to generate new tree constraints and update G*. To update G*, if more than one connected component is combined into a new one by new tree constraints, we arbitrarily choose one of the old seeds from these connected components as a new seed, and perform a graph traversal to update *W*-values within the new connected component. A non-tree edge that fails to receive a synthetic cycle in a trial of cycle synthesis may benefit from a later updated G* and therefore our extension procedure is designed to operate in an iterative fashion; the procedure terminates only if G*cannot be updated anymore. In this procedure, a non-tree edge may be checked many times (in different iterations) to form a synthetic cycle. In the worst case scenario, only one cycle is synthesized in each iteration, so we require *k *iterations to perform *k *+ (*k *- 1) + ... + 1 = *O*(*k*^2^) trials of cycle synthesis.

To verify the correctness of the extension procedure, we need first to explain the meaning of Equation (4). Follow a similar argument to that of Lemma 1, for two nodes *n_x _*and *n_y _*that reside in the same connected component of G*, we know that *W*[*n_x_*] + *W*[*n_y_*] is actually the tree constraint of the path from *n_x _*to *n_y _*on *T*(*G*). The synthetic cycle is conceptually a round trip through tree edges and the non-tree edge *e*. The value $\hat{b_{c}}$ in Equation (4) is therefore the summation of *h*-variables along the round trip (Figure 5(b)). Now we demonstrate that $\hat{b_{c}}$ is the same as the cycle constraint of *e*. We first show that there is exactly one *h*-variable of *e *in $\hat{b_{c}}$. According to Equation (4), we have 2*t *+ 1 *h*-variables of *e *in $\hat{b_{c}}$. Since we perform additions over GF(2), 2*t *out of the 2*t *+ 1 *h*-variables will be cancelled and we finally have only one *h*-variable of *e *in $\hat{b_{c}}$. To verify if $\hat{b_{c}}$is the same as the cycle constraint of *e *in *G*, we assume that the expected cycle constraint of *e *is *b_c_*. We generate a set $S_{e}^{\prime }$ by converting path constraints (*n_i_, n_j_, b_p_, e*) in *S_e _*to tree constraints (*n_i_, n_j_, b_c _*+ *b_p_*). It is easy to see that the converted 2*t *+ 1 tree constraints also link connected components in G* to form a new synthetic cycle, and the corresponding round trip only contains tree edges in *T*(*G*). *T*(*G*) has no cycle and therefore each edge of this new round trip must be visited an even number of times, which means that its *h*-variable will be cancelled in the new cycle constraint. So the constraint of the new synthetic cycle must be zero and we have the following equations:

$$\underset{\left(n_{i},n_{j},b_{c}+b_{p}\right)\in S_{e}^{\prime }}{\sum }W\left[n_{i}\right]+W\left[n_{j}\right]+b_{p}+b_{c}=\hat{b_{c}}+\overset{\left|S_{e}^{\prime }\right|}{\underset{i=1}{\sum }}b_{c}=0.$$

Since there are 2*t *+ 1 constraints in $S_{e}^{\prime }$, we have $\sum_{i=1}^{\left|S_{e}^{\prime }\right|}b_{c}=b_{c}$. We then obtain $\hat{b_{c}}+b_{c}=0$ and conclude that $\hat{b_{c}}=b_{c}$.

For each *e *∈ *E^S^*, the time to determine if there are odd number of path constraints that link connected components in G* to form a cycle is *O*(*m*). This time complexity can be achieved by regarding each connected component as a single node and each path constraint of *e *as a single edge, and following *O*(*m*) edges to perform a depth-first traversal. Since there are *O*(*k*^2^) cycle syntheses throughout the extension procedure, we require *O*(*k*^2^*m*) time to find synthetic cycles. Once we synthesized a cycle for *e*, we require *O*(*m*) time to convert path constraints to tree constraints because there are at most *m *path constraints of *e *in *C^P^*. There are *O*(*k*) non-tree edges in *E^S ^*and therefore the extension procedure takes *O*(*km*) time to perform constraint conversion. To update G*, we require *O*(*n*) time to perform breadth-first traversal on every connected component to modify *W*-values similar to the way we initialize G*. There are at most *k *synthetic cycles and therefore G* is updated *O*(*k*) times in *O*(*kn*) time. In summary, the Step 3, *constraint reduction and transformation*, takes *O*(*k*^2^*m*) + *O*(*km*) + *O*(*kn*) = *O*(*k*^2^*m *+ *kn*) time.

#### Step 4: haplotype determination

To solve the *h*-values of the tree edges of *T*(*G*) by Lemma 2, we try to make G* produced by Step 3 connected. Firstly, we pay attention to the founders in the pedigree. Founders cannot be predetermined endpoints of paths with either path constraints or tree constraints and therefore founders must be isolated nodes in G*. It is also impossible to know whether an allele of a founder is paternal or maternal. We attach a founder *n_f _*to G* by assuming that it passed its paternal haplotype to an arbitrary child *n_c_*. The attachment can be done by assigning weight zero to the edge (*n_f_, n_c_*) of G*, which implies $h_{n_{f},n_{c}}=0$ (*n_f _*passes its paternal haplotype). There are *O*(*n*) edges in G* and therefore the attachment of founders to G* takes *O*(*n*) time.

Secondly, we check if there is any non-tree edge that can link any two connected components of G*. A non-tree edge *e *= (*n_i_, n_j_*) can link two connected components *A *and *B *if we can find a path constraint (*n_k_, n_l_, b_p_, e*) of path *p *that, without loss of generality, satisfies the following two conditions:

1. *n_k _*and *n_i _*reside in *A *and have available *W *[*n_k_*] and *W *[*n_i_*] derived from the seed *n_A _*of *A*,

2. *n_l _*and *n_j _*reside in *B *and have available *W *[*n_l_*] and *W *[*n_j_*] derived from the seed *n_B _*of *B*.

If we can find such a non-tree edge *e*, we can decompose *p *into three parts: a sub-path from *n_k _*to *n_i_*, the non-tree edge *e*, and the sub-path from *n_j _*to *n_l_*. The constraints of these three parts are $W\left[n_{k}\right]+W\left[n_{i}\right],\;h_{n_{i},n_{j}},W\left[n_{j}\right]+W\left[n_{l}\right]$, respectively. This turns out that $b_{p}=W\left[n_{k}\right]+W\left[n_{i}\right]+h_{n_{i},n_{j}}+W\left[n_{j}\right]+W\left[n_{l}\right]$. The non-tree edge *e *therefore can be used to link components *A *and *B *with known *h*-value $h_{n_{i},n_{j}}=b_{p}+W\left[n_{k}\right]+W\left[n_{i}\right]+W\left[n_{j}\right]+W\left[n_{l}\right]$. Since there are at most *O*(*mn*) path constraints to be checked, this procedure requires *O*(*mn*) time.

Finally, assume that there remain *t *connected components of G*. We arbitrarily introduce (*t *-1) edges into G* to make it connected. Our algorithm does not impose any constraint on these (*t *- 1) edges and therefore the weights of these edges can be safely set as free variables. We then update all *W*-values within the new connected G* (new *W*-values may contain free variables), and apply Lemma 2 to determine the *h*-values of all edges in *T*(*G*). With these solved *h*-values as well as the *w*-constants and *d*-constants, we can determine the *p*-values of all nodes in the locus graphs by Equation (1).

The update of G* takes *O*(*n*) time. Moreover, we require *O*(*n*) time to determine all *h*-values of edges in *T*(*G*). For a locus graph, the determined *h*-values are used to solve all *p*-values in *O*(*n*) time. Since there are *m *locus graphs, we require *O*(*mn*) time to determine the *p*-vectors of all nodes in *G*. Consequently, the three procedures of this step take *O*(*n*) + *O*(*mn*) + *O*(*n*) + *O*(*mn*) = *O*(*mn*) time.

## Results and discussion

### An execution example

**Figure 6:**
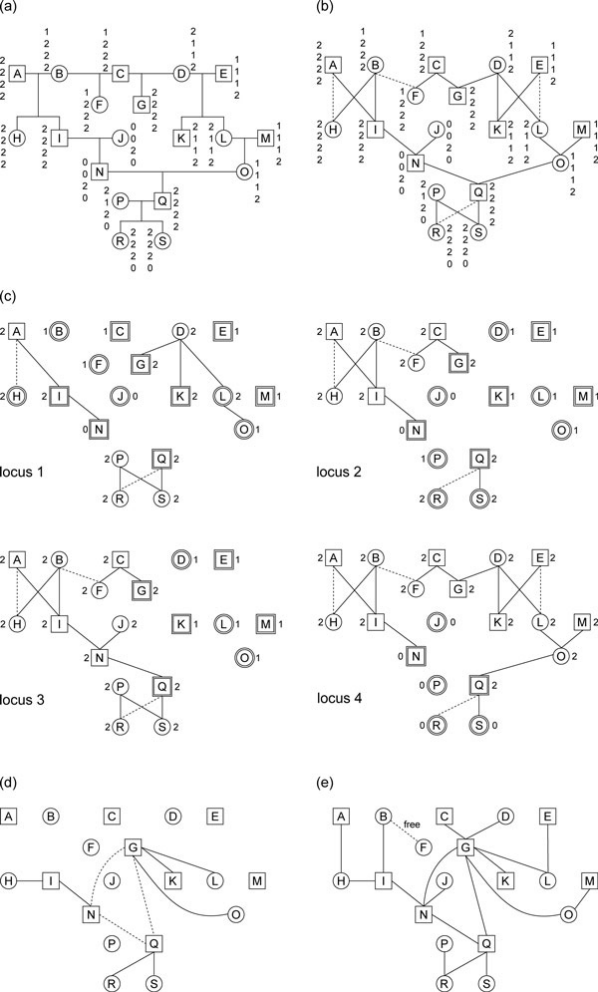
**An execution example**. (a) A pedigree of 19 individuals with genotype data. (b) The corresponding pedigree graph *G *with a spanning tree *T*(*G*). Tree edges are solid lines and non-tree edges are dotted lines. (c) Locus graphs and forests. Nodes with thick borders are predetermined. (d) The corresponding constraint graph G*. Nodes and solid lines compose the initial constraint graph. The three dotted lines are path constraints that form a synthetic cycle of the non-tree edge B-F. (e) The final G*. All edges except B-F have weight zero; *h*_*B, F *_is a free variable.

We use the pedigree given in Figure 6(a) as an example to demonstrate how the proposed algorithm works. There are 19 individuals in the pedigree; eight of them are founders. Each individual is equipped with genotype data collected from four marker loci. There is a mating loop in the pedigree.

In the first step, we transform the input pedigree into a pedigree graph *G *and construct a spanning tree *T*(*G*) on *G *(Figure 6(b)). There are three local cycles A-H-B-I-A, D-K-E-L-D, and P-R-Q-S-P and one global cycle B-F-C-G-D-L-O-Q-N-I-B in *G*. Edges A-H, E-L, Q-R, and B-F are chosen as non-tree edges within the four cycles. From the pedigree graph *G*, we construct the four locus graphs and forests that are depicted in Figure 6(c). The *p*-values of predetermined nodes, *w*-constants of all nodes, and *d*-constants of all edges within the four locus graphs are also identified.

In the second step, we generate all cycle, path, and tree constraints for each of the four locus graphs using Equations (2) and (3). For example, cycle A-H-B-I-A in the second locus graph has cycle constraint *h_A, H _*+ *h_H, B _*+*h_B, I _*+*h_I, A _*= *d_A, H _*[2] + *d_H, B _*[2] +*d_B, I _*[2] +*d*_*I, A *_[2] = 0 + 1 + 1 + 0 = 0, and path G-C-F-B-I-N-Q in the third locus graph has path constraint of the non-tree edge B-F *h_G, C _*+ *h_C, F _*+ *h_F, B _*+ *h_B, I _*+ *h_I, N _*+ *h_N, Q _*= *p_G_*[3] + *d_G, C_*[3] + *d_C, F _*[3] + *d_F, B_*[3] + *d_B, I _*[3] + *d_I, N _*[3] + *d_N, Q_*[3] + *p_Q_*[3] = 0 + 0 + 0 + 1 + 1 + 0 + 0 + 0 = 0.

At the end of this step we receive *C^C ^*= {(0, *e_E-L_*), (0, *e_Q-R_*), (0, *e_A-H_*)}, *C^P ^*= {(*I, H*, 0, *e_A-H_*), (*N, G*, 0, *e_B-F)_*, (*G, Q*, 0, *e_B-F)_*, (*R, Q*, 0, *e_R-Q_*), (*N, Q*, 0, *e_B-F)_*}, and *C_T _*= {(*I, N*, 0), (*G, K*, 0), (*G, L*, 0), (*G, O*, 0), (*Q, S*, 0)}.

In the third step, we obtain two new tree constraints (*R, Q*, 0) and (*I, H*, 0) by (0, *e_Q-R_*) + (*R, Q*, 0, *e_R-Q_*) and (0, *e_A-H_*) + (*I, H*, 0, *e_A-H_*), respectively. The set *C^T ^*is therefore extended to {(*I, N*, 0), (*G, K*, 0), (*G, L*, 0), (*G, O*, 0), (*Q, S*, 0), (*R, Q*, 0), (*I, H*, 0)}. We construct the initial constraint graph G* based on the updated *C^T ^*(Figure 6(d)). In the initial G*, we choose H, G, and Q as component seeds to determine *W*-values. We can further find that the three path constraints (*N, G*, 0, *e*_*B-F*)_,(*G, Q*, 0, *e*_*B-F*)_, and (*N, Q*, 0, *e*_*B-F*) _link three connected components to form a synthetic cycle of the non-tree edge B-F with constraint zero. So we further obtain three extra tree constraints (*N, G*, 0), (*G, Q*, 0), and (*N, Q*, 0) derived from the synthetic cycle and add them to G*.

In the final step, we try to make G* connected to solve all *h*-values. We first arbitrarily introduce eight edges A-H, B-I, C-G, D-G, E-L, J-N, M-O, and P-R to attach the eight founders to G*; all the eight edges are of weight zero to imply that founders passes their paternal haplotypes to one of their children. Now there are only two connected components in G*, one of which is an isolated node, F. we attach F to G* by set *h_B, F _*as a free variable. This final connected G* is depicted in Figure 6(e). After the final update of G*, all *h*-values other than *h*_*B, F *_are zero, and *h_B, F _*is free to be either zero or one. Given these known *h*-values, all *p*-values over the four locus graphs can be solved by Equation (1).

### Time complexity and experimental result

According to the analyses at the end of each step in Section 3, the time complexity of our algorithm is *O*(*mn*) (step 1: preprocessing) + *O*(*kmn*) (step 2: constraint generation) + *O*(*k*^2^*m *+ *kn*) (step 3: constraint reduction and transformation) + *O*(*mn*) (step 4: haplotype determination) = *O*(*kmn *+ *k*^2^*m*). Because *k *is regarded as a constant, our algorithm is linear.

To verify the efficiency and the correctness of our algorithm, we conducted some experiments using the proposed method. Our algorithm was implemented in C and was evaluated on a desktop computer equipped with Intel Core i7-2600 3.4 GHz CPU and 8 GB of RAM. The desktop ran Ubuntu Release 11.10 operating system with Linux kernel 3.0.0-16-generic and GNOME 3.2.1 graphical user interface.

In the experiments, we generated test cases by setting different number of individuals (*n*) and markers (*m*). We applied the algorithm developed by Thomas *et al*. [24] to generate 12 tree pedigrees with different *n *values ranging from 30 to 400. To observe how the number of mating loops (*k*) affects our algorithm, each tree pedigree was preprocessed to produce four variants with zero, two, four, and six mating loops. For each pedigree, we examined 10 different *m *values ranging from 10 to 300. Each (*n, m*) combination was tested 100 times. Each time we generated new genotypes and randomly selected one pedigree from the four variants of the given *n*. The haplotype configurations of all the 12000 trials were correctly identified. The experimental results are listed in Table 2.

**Table 2:** Experimental results

						Number of individuals (*n*)							
		
Number of loci (*m*)		30	60	100	130	160	200	230	260	300	330	360	400
	10	0.02	0.00	0.00	0.02	0.05	0.01	0.02	0.01	0.04	0.03	0.02	0.02
	30	0.00	0.00	0.00	0.00	0.00	0.00	0.00	0.00	0.00	0.00	0.00	0.00
	60	0.00	0.00	0.00	0.00	0.00	0.00	0.00	0.00	0.00	0.00	0.00	0.00
	100	0.00	0.00	0.00	0.00	0.00	0.00	0.00	0.00	0.00	0.00	0.00	0.00
	130	0.00	0.00	0.00	0.00	0.00	0.00	0.00	0.00	0.00	0.00	0.00	0.00
(a)	160	0.00	0.00	0.00	0.00	0.00	0.00	0.00	0.00	0.00	0.00	0.00	0.00
	200	0.00	0.00	0.00	0.00	0.00	0.00	0.00	0.00	0.00	0.00	0.00	0.00
	230	0.00	0.00	0.00	0.00	0.00	0.00	0.00	0.00	0.00	0.00	0.00	0.00
	260	0.00	0.00	0.00	0.00	0.00	0.00	0.00	0.00	0.00	0.00	0.00	0.00
	300	0.00	0.00	0.00	0.00	0.00	0.00	0.00	0.00	0.00	0.00	0.00	0.00

	10	0.10	0.40	0.55	0.50	0.51	0.61	0.99	0.52	1.41	1.77	1.28	0.41
	30	0.11	0.50	0.90	0.48	0.66	0.55	2.28	1.22	1.06	2.94	1.16	2.71
	60	0.21	1.66	1.09	0.68	2.19	2.77	1.63	3.26	3.08	2.76	2.20	4.38
	100	0.49	1.03	1.79	1.63	1.93	2.66	3.21	3.42	3.63	4.65	3.42	3.73
	130	1.74	1.55	2.39	1.24	1.84	3.57	3.07	3.49	4.81	3.38	5.01	5.26
(b)	160	0.57	1.53	2.17	1.53	2.99	3.76	3.71	4.81	6.44	4.52	5.99	6.45
	200	1.20	1.82	2.02	2.10	5.18	4.31	4.89	4.49	5.37	6.16	6.77	8.87
	230	0.70	2.59	2.34	2.71	5.52	3.79	5.28	6.19	6.63	7.89	7.87	9.77
	260	1.15	2.29	2.62	3.72	5.10	5.99	5.97	6.45	7.12	8.34	10.11	10.77
	300	1.67	2.31	3.33	4.27	5.27	6.49	6.35	7.11	8.70	9.12	11.40	13.22

	10	3.52	3.36	2.92	3.18	3.02	3.18	3.36	3.08	2.98	2.42	3.06	3.14
	30	2.96	3.00	2.98	3.14	3.18	2.90	2.96	3.04	3.18	3.02	3.22	2.94
	60	3.08	3.08	3.02	2.66	3.36	2.92	3.24	3.02	3.10	2.86	2.90	2.86
	100	3.00	2.90	2.78	3.36	3.00	3.28	3.38	2.72	3.30	2.66	2.98	2.74
	130	2.80	3.00	3.24	3.50	3.72	3.30	2.90	3.04	3.08	2.94	3.68	3.20
(c)	160	3.02	3.18	3.46	2.92	3.10	2.86	3.32	3.40	2.88	3.40	3.16	2.62
	200	2.84	3.18	3.06	2.76	2.78	2.82	3.14	3.12	3.12	2.86	3.00	3.14
	230	3.24	3.22	2.90	2.74	3.32	2.86	2.94	3.34	3.08	2.70	2.84	3.42
	260	2.72	2.70	2.66	3.00	3.22	3.42	3.10	3.32	3.24	2.86	2.66	2.92
	300	3.24	3.02	2.70	2.76	2.92	2.74	2.94	2.98	2.62	3.02	3.34	3.44

(a) Average number of free variables. (b) Execution time (seconds) to generate solutions. Each entry in the table is the cumulative execution time of 100 replicates. (c) Average number of mating loops.

Table 2(a) shows that unknown *p*-variables were correctly solved without assigning any free variable if the number of marker loci was not less than 30, which covers most practical cases in regular genotyping. Free variables were required only when the number of marker loci was far less than the number of individuals. In this experiment, free variables were used only when *m *= 10, and they were used at most five times out of 100 trials. The result is reasonable because the dimension of the solution space of a pedigree with a limited number of marker loci is probable less than the number of unknown *p*-variables.

Table 2(b) shows the cumulative execution time of 100 trials of each (*n, m*) combination. We received a fluctuation in execution time if *n *or *m *were less than 100. We conjecture that, because the algorithm executes very fast for small values of *n *or *m*, the cumulative execution time might be dramatically affected by the context switches within the operating system that ran many background services. Furthermore, we believe that when both *n *and *m *were larger than 100, the execution time of the algorithm became more significant than that of the context switches. From the table it is apparent that the execution time is linear for the larger *n *and *m *values.

Finally, Table 2(c) shows that mating loops existed evenly throughout all 12000 trials, with the number ranging from zero to six per pedigree, and the number did not affect the linearity of the execution time of our algorithm in relation to the input size of *n *and *m*.

### Issue of spanning tree and seed node selection

In the first step, *preprocessing*, a spanning tree *T*(*G*) is constructed on the pedigree graph *G*. As mentioned above, *T*(*G*) is constructed for the ease of cycle processing; it is merely an auxiliary data structure used to generate linear constraints of all cycles and paths between predetermined nodes in *G*. We do not impose any constraint on the construction of *T*(*G*) because predetermined nodes are defined by genotype data. Once the input pedigree is given, all the cycles and paths as well as their constraints are bound, no matter which spanning tree is constructed on the pedigree graph. Different spanning trees assign different edges as the non-tree edge in a cycle, and only affect the type of a constraint; a constraint may be a path constraint with respective to one spanning tree and a tree constraint with respect to another spanning tree. Since different spanning trees are used to generate the same set of constraints, without considering their type, the construction of the spanning tree can be arbitrary. In our implementation, *T*(*G*) was constructed by depth-first traversal.

In the second step, *constraint generation*, a seed node is arbitrarily selected from *T*(*G*) to generate tree constraints. To see why the seed node can be selected arbitrarily, assume that there are two possible seeds *n_i _*and *n_j_*. For any other predetermined node *n_k_*, we have (*n_j_, n_k_, b_jk_*) = (*n_i_, n_j_, b_jk_*) + (*n_i_, n_k_, b_ik_*) by Lemma 1, which means that a tree constraint seeded with one predetermined node is a linear combination of two tree constraints seeded with another predetermined node. Hence, tree constraints seeded with different predetermined nodes are mathematical equivalent; we can safely choose any predetermined node as seed. Similarily, the seed nodes within a constraint graph can also be selected arbitrarily based on the above argument.

### Consistency checking

Although we assume that the input pedigree is free of genotyping errors, our algorithm can be easily modified to detect inconsistencies within the genotype data without loss of efficiency. No recombination is allowed in the input pedigree and therefore inconsistencies will arise if there are different assignments of an *h*-value, that results in incompatible linear constraints. We may designate the following two checkpoints to detect inconsistencies within our linear system:

1. *The generation of constraints*. The constraint of a path or a cycle may be computed more than one time across all locus graphs; all these computations should arrive at the same value. So each time we compute a constraint, we check if it is the same as the current value, if any.

2. The initialization/update of G*. There may be loops in the constraint graph G* and therefore it is possible that there are more than one path from the seed *n_s _*to a node *n_i_*. It turns out that *W *[*n_i_*] may be assigned more than once in the initialization or update procedures of G*. By the definition of *W*-variables, however, all the assignments to *W*[*n_i_*] are actually associated with the same path from *n_s _*to *n_i _*on *T *(*G*) and therefore should be identical. So each time we compute a *W*-value, we check if it agrees with the current value, if any.

## Conclusions

In this study, we proposed and implemented an algorithm to solve the zero-recombinant haplotype configuration (ZRHC) problem for a general pedigree in *O*(*kmn *+ *k*^2^*m*) time. With the aid of free variables, our method provides a general solution to describe possible haplotype structures within a pedigree rather than a particular solution that only assigns a specific numerical setting to haplotypes. To the best of our knowledge, this algorithm is the first deterministic one to provide a general solution in linear time for pedigrees having small number of mating loops. Moreover, the algorithm can be easily modified to detect inconsistency among genotype data without loss of efficiency. Our experimental results confirm its linearity. In the future, we will try to extend the proposed algorithm to handle recombination and missing data in linear time for general pedigrees.

## Competing interests

The authors declare that they have no competing interests.

## Authors' contributions

EYL, WBW, TJ, and KPW contributed to the algorithm design. EYL implemented the algorithms and performed the experiments. KPW and EYL analyzed the complexity of the algorithm and wrote the paper. All authors read and approved the final manuscript.

## References

[B1] Qian D Beckmann L Minimum-recombinant haplotyping in pedigrees The American Journal of Human Genetics 2002 70 6 1434 1445 10.1086/340610 PMC379131 11992251

[B2] Albers CA Heskes T Kappen HJ Haplotype inference in general pedigrees using the cluster variation method Genetics 2007 177 2 1101 1116 10.1534/genetics.107.074047 17660564 PMC2034616

[B3] Chin FYL Zhang Q Shen H *k*-recombination haplotype inference in pedigrees Proceedings of the International Conference on Computational Science (ICCS) 2005 Springer-Verlag, Berlin 985 993

[B4] Li J Jiang T Efficient rule-based haplotyping algorithms for pedigree data Proceedings of the 7th Annual Conference on Research in Computational Molecular Biology (RECOMB) 2003 ACM, New York 197 206

[B5] Li J Jiang T An exact solution for finding minimum recombinant haplotype configurations on pedigrees with missing data by integer linear programming Proceedings of the 8th Annual Conference on Research in Computational Molecular Biology (RECOMB) 2004 ACM, New York 20 29

[B6] Li J Jiang T Computing the minimum recombinant haplotype configuration from incomplete genotype data on a pedigree by integer linear programming Journal of Computational Biology 2005 12 6 719 739 10.1089/cmb.2005.12.719 16108713

[B7] Sobel E Lange K O'Connell JR Weeks DE Speed T, Waterman MS Haplotyping algorithms Genetic Mapping and DNA Sequencing, Volume 81 of IMA Volumes in Mathematics and its Applications 1996 Springer-Verlag 89 110

[B8] Tapadar P Ghosh S Majumder PP Haplotyping in pedigrees via a genetic algorithm Human Heredity 2000 50 43 56 10.1159/000022890 10545757

[B9] O'Connell JR Zero-recombinant haplotyping: Applications to fine mapping using SNPs Genetic Epidemiology 2000 19 64 70 10.1002/1098-2272(200007)19:1<64::AID-GEPI5>3.0.CO;2-E 11055372

[B10] Chan MY Chan WT Chin FYL Fung SPY Kao MY Linear-time haplotype inference on pedigrees without recombinations and mating loops SIAM J Comput 2009 38 6 2179 2197 10.1137/080680990

[B11] Li X Li J Efficient haplotype inference from pedigrees with missing data using linear systems with disjoint-set data structure7th Annual International conference on Computational Systems Bioinformatics 2008 297 308 PMC3326667 19642289

[B12] Liu L Jiang T A linear-time algorithm for reconstructing zero-recombinant haplotype configuration on pedigrees without mating loops Journal of Combinatorial Optimization 2008 19 217 240

[B13] Baruch E Weller JI Cohen-Zinder M Ron M Seroussi E Efficient inference of haplotypes from genotypes on a large animal pedigree Genetics 2006 172 3 1757 1765 16361242 10.1534/genetics.105.047134 PMC1456282

[B14] Doan DD Evans PA Horton JD A near-linear time algorithm for haplotype determination on general pedigrees J Comput Biol 2010 17 10 1451 65 http://www.biomedsearch.com/nih/near-linear-time-algorithm-haplotype/20937017.html 10.1089/cmb.2009.0133 20937017

[B15] Wang WB Jiang T Kucherov G, Ukkonen E Efficient inference of haplotypes from genotypes on a pedigree with mutations and missing alleles CPM 2009, LNCS 5577 2009 Springer-Verlag Berlin Heidelberg 353 367

[B16] Xiao J Liu L Xia L Jiang T Efficient algorithms for reconstructing zero-recombinant haplotypes on a pedigree based on fast elimination of redundant linear equations SIAM J Comput 2009 38 2198 2219 10.1137/070687591

[B17] Zhang K Sun F Zhao H HAPLORE: a program for haplotype reconstruction in general pedigrees without recombination Bioinformatics 2005 21 90 103 10.1093/bioinformatics/bth388 15231536

[B18] Gusfield D Inference of haplotypes from samples of diploid populations: complexity and algorithms Journal of Computational Biology 2001 8 3 305 323 10.1089/10665270152530863 11535178

[B19] Niu T Qin ZS Xu X Liu JS Bayesian haplotype inference for multiple linked single-nucleotide polymorphisms The American Journal of Human Genetics 2002 70 157 169 10.1086/338446 PMC448439 11741196

[B20] Stephens M Smith NJ Donnelly P A new statistical method for haplotype reconstruction from population data The American Journal of Human Genetics 2001 68 4 978 989 10.1086/319501 PMC1275651 11254454

[B21] Wang S Kidd KK Zhao H On the use of DNA pooling to estimate haplotype frequencies Genetic Epidemiology 2003 24 74 82 10.1002/gepi.10195 12508258

[B22] Yang Y Zhang J Hoh J Matsuda F Xu P Lathrop M Ott J Efficiency of single-nucleotide polymorphism haplotype estimation from pooled DNA Proceedings of the National Academy of Science of the United States of America 2002 100 7225 7230 10.1073/pnas.1237858100 PMC165857 12777616

[B23] Browning SR Browning BL Haplotype phasing: existing methods and new developments Nature Reviews Genetics 2011 12 703 714 http://www.nature.com/nrg/journal/v12/n10/full/nrg3054.html 10.1038/nrg3054 PMC3217888 21921926

[B24] Thomas A Cannings C Simulating realistic zero loop pedigrees using a bipartite Prufer code and graphical modelling Math Med Biol 2004 21 4 335 45 http://www.biomedsearch.com/nih/Simulating-realistic-zero-loop-pedigrees/15567888.html 10.1093/imammb/21.4.335 15567888

